# Profile of differentially expressed genes mediated by the type III epidermal growth factor receptor mutation expressed in a small-cell lung cancer cell line

**DOI:** 10.1054/bjoc.2001.2053

**Published:** 2001-10

**Authors:** M W Pedersen, T Thykjær, T F Ørntoft, L Damstrup, H S Poulsen

**Affiliations:** Department of Radiation Biology, The Finsen Centre, National University Hospital, Section 6321, Copenhagen, DK-2100, Denmark; Department of Clinical Biochemistry, Skejby Hospital, Århus, Denmark

**Keywords:** EGFRvIII, profile, gene, expression

## Abstract

Previous studies have shown a correlation between expression of the EGF receptor type III mutation (EGFRvIII) and a more malignant phenotype of various cancers including: non-small-cell lung cancer, glioblastoma multiforme, prostate cancer and breast cancer. Thus, a detailed molecular genetic understanding of how the EGFRvIII contributes to the malignant phenotype is of major importance for future therapy. The GeneChip Hu6800Set developed by Affymetrix was used to identify changes in gene expression caused by the expression of EGFRvIII. The cell line selected for the study was an EGF receptor negative small-cell-lung cancer cell line, GLC3, stably transfected with the EGFRvIII gene in a Tet-On system. By comparison of mRNA levels in EGFRvIII-GLC3 with those of Tet-On-GLC3, it was found that the levels of mRNAs encoding several transcription factors (ATF-3, JunD, and c-Myb), cell adhesion molecules (CD36, CD24), signal transduction related molecules (MKP-1) and other molecules related to cancer (CD98, thymosin beta-10) were altered in the EGFRvIII transfected cell line. Northern hybridisations and Western blot analyses were used to verify selected results. The results indicate that expression of EGFRvIII alters expression of genes involved in the control of cell growth, survival and motility. © 2001 Cancer Research Campaign  http://www.bjcancer.com


*British Journal of Cancer* (2001) **85**(8), 1211–1218
© 2001 Cancer Research Campaign
doi: 10.1054/ bjoc.2001.2053, available online at http://www.idealibrary.com on IDEAL

### Profile of differentially expressed genes mediated by the type III epidermal growth factor receptor mutation expressed in a small-cell lung cancer cell line

**MW Pedersen[1], T Thykjær[2], TF Ørntoft[2], L Damstrup[1] and HS Poulsen[1]**

[1]Department of Radiation Biology, The Finsen Centre, Section 6321, National University Hospital, DK-2100 Copenhagen, Denmark
[2]Department of Clinical Biochemistry, Skejby Hospital, Århus, Denmark

**Summary** Previous studies have shown a correlation between expression of the EGF receptor type III mutation (EGFRvIII) and a more malignant phenotype of various cancers including: non-small-cell lung cancer, glioblastoma multiforme, prostate cancer and breast cancer. Thus, a detailed molecular genetic understanding of how the EGFRvIII contributes to the malignant phenotype is of major importance for future therapy. The GeneChip Hu6800Set developed by Affymetrix was used to identify changes in gene expression caused by the expression of EGFRvIII. The cell line selected for the study was an EGF receptor negative small-cell-lung cancer cell line, GLC3, stably transfected with the EGFRvIII gene in a Tet-On system. By comparison of mRNA levels in EGFRvIII-GLC3 with those of Tet-On-GLC3, it was found that the levels of mRNAs encoding several transcription factors (ATF-3, JunD, and c-Myb), cell adhesion molecules (CD36, CD24), signal transduction related molecules (MKP-1) and other molecules related to cancer (CD98, thymosin beta-10) were altered in the EGFRvIII transfected cell line. Northern hybridisations and Western blot analyses were used to verify selected results. The results indicate that expression of EGFRvIII alters expression of genes involved in the control of cell growth, survival and motility. © 2001 Cancer Research Campaign http://www.bjcancer.com

**Keywords**: EGFRvIII; profile; gene; expression

The epidermal growth factor receptor (EGFR or ErbB-1) is a 170 kDa cell surface glycoprotein consisting of a single polypeptide chain of 1186 amino acid residues. EGFR binds 6 ligands: EGF, TGF-α, amphiregulin, heparin-binding EGF-like growth factor, epiregulin and betacellulin and plays an important role in the regulation of normal cell growth, proliferation and differentiation (Prigent and Lemoine, 1992).

In a large number of tumours the EGFR status is altered due to overexpression, amplification and/or mutations in the *EGFR* gene. A significant fraction of amplified *EGFR* genes are rearranged to specifically eliminate a DNA fragment containing exons 2–7 of the gene resulting in an in-frame deletion of 801 base pairs of the extracellular domain encoding sequence. This rearrangement is designated as the type III EGFR mutation (EGFRvIII, ΔEGFR, de2-7 EGFR) and is the most common mutation in the *EGFR* gene. The EGFRvIII has been detected in 16% of non-small-cell lung cancer (NSCLC), 57% of high-grade glial tumours, 86% of low-grade gliomas, 66% of paediatric gliomas, 86% of medulloblastomas, 78% of breast carcinomas, and 73% of ovarian carcinomas (Garcia et al, 1993; Moscatello et al, 1995). In prostate cancer expression of EGFRvIII appears to increase in a correlated manner with the gradual transformation of the tissues to the malignant phenotype (Olapade-Olaopa et al, 2000). This high prevalence in a wide variety of human tumours suggests that it is positively selected during the process of tumorigenesis due to enhancement of the tumour-inducing properties of the EGFR. Several studies support this hypothesis and strongly indicate a correlation between the expression of EGFRvIII and an enhanced tumorigenicity of tumour cells both in vitro and in vivo (Nishikawa et al, 1994; Batra et al, 1995; Nagane et al, 1996, 1998; Sugawa et al, 1998). Although EGFRvIII is not able to bind EGF or any of the other EGFR ligands, it is constitutively phosphorylated and able to activate downstream signalling cascades. However, the EGFRvIII seems to utilize a different downstream signalling network as compared to the ligand-activated EGFR. The Ras/Raf/ERK pathway appears to be down-regulated by EGFRvIII, while a pathway composed of phosphatidyl inositol 3 kinase (P13K), and c-Jun N-terminal Kinase (JNK) is constitutively active (Antonyak et al, 1998; Moscatello et al, 1998). This difference in signalling between EGFRvIII and EGFR may, at least partly, explain the increased tumorgenicity of EGFRvIII-expressing cells.

Very little is known regarding genes whose expression is regulated by EGFRvIII and its downstream-signalling cascade. For the first time, we set out to identify EGFRvIII-regulated genes. Affymetrix' oligonucleotide array Hu6800set, containing probes for more than 6800 human genes, was used to assay for changes in mRNA expression between an EGFRvIII transfected human small-cell lung cancer cell line, GLC3-EGFRvIII, and a vector control cell line. Amongst the 6800 genes analysed, we found 225 whose expression was altered by at least a factor of 2. These included genes encoding adhesion molecules, transcription factors, and signal-modulating molecules. Northern blot and Western blot analyses were used to confirm selected results.

*Received 22 January 2001*
*Revised 11 June 2001*
*Accepted 20 June 2001*

*Correspondence to:* HS Poulsen
E-mail: skovgaard@rh.dk

Supported by the Danish Cancer Society and the Danish Research Council.

### MATERIALS AND METHODS

### Materials

Antibodies to MKP-1 and CD98 were obtained from Santa-Cruz Biotechnology (Santa-Cruz, CA, USA). Precast SDS-PAGE gels were from Invitrogen (The Nederlands). Platinum Taq polymerase was from Life Technologies (Scotland). All chemicals were purchased from Sigma-Aldrich (Denmark) unless otherwise stated.

### Cell culture and treatment

The EGFRvIII-expressing human small-cell lung cancer cell lines has been described by Damstrup et al and was generated by transfecting the human, EGFR-negative small-cell lung cancer cell line, GLC3, with EGFRvIII in a Tet-On system. The transfected cells were found to be more invasive in vitro and to have a lower number of junctional complexes as compared to the vector controls.[1] Cell lines were cultured at 37˚C in humidified 5% $CO_2$ in RPMI 1640 medium (Life Technologies, Scotland) supplemented with 10% heat-inactivated tet-approved fetal calf serum (Clontech, UK). EGFRvIII expression in the transfected cells was induced by addition of 2 $\mu$g ml$^{-1}$ doxycycline. The control cells were treated simultaneously with the same concentration of doxycycline.

### Biotinylated probes preparation

Total RNA was isolated from $10^8$ cells using Trizol reagent (Life Technologies, UK). 10 $\mu$g of total RNA pooled from 4 independent samples was used as starting material for cDNA preparation. Double-stranded cDNA was prepared using Life Technologies (UK) Superscript Choice system™ according to manufacturer's instructions using an oligo$(dT)_{24}$-anchored T7 primer. Biotinylated RNA was synthesized using the T7 MEGAscript in vitro transcription kit (Ambion Inc, TX, USA) with biotin-11-CTP and biotin 16-UTP (Enzo, NY, USA) for 4 h at 37˚C. In vitro transcription products were purified using RNeasy columns from Qiagen (UK).

### Array Hybridization and Scanning

10 $\mu$g of the biotinylated RNA was fragmented for 35 min at 94˚C in a buffer composed of 40 nM Tris-acetate, pH 8.1, 100 nM KOAc, and 30 nM MgOAc. Prior to hybridisation, the fragmented cRNA was heated to 95˚C for 5 min in a 6 × SSPE-T hybridisation buffer (1 M NaCl, 10 mM Tris pH 7.6, 0.005% Triton) and subsequently at 40˚C for 5 min before loading onto the Affymetrix probe array cartridge. The probe arrays were incubated for 16 h at 40˚C at constant rotation (60 rpm). Fluidic station 400 from Affymetrix was used for washing and staining of the arrays.

The probe array was washed 10 times in 6 × SSPE-T at 25˚C followed by 4 washes in 0.5 × SSPE-T at 50˚C. A 3-step protocol was used for detection of the hybridised biotinylated RNA: first the probe array was incubated with a streptavidin- phycoerythrin conjugate, 10 $\mu$g ml$^{-1}$ (Molecular Probes, OR, USA) in 6 × SSPE-T for 30 min at 25˚C followed by labelling with an anti-streptavidin goat biotinylated antibody (Vector Laboratories, Burlingham, CA, USA) and a final staining again with the streptavidin-phycoerythrin conjugate.

The Probe arrays were scanned at 560 nm using a confocal laser scanner with an argon ion laser (Hewlett Packard GeneArray Scanner G2500A). The digitalized image data were processed using the Affymetrix Gene Expression Analysis Software (version 3.1). For comparison between chips, they were scaled to a global intensity of 150, as previously published (Der et al, 1998; Kaminski et al, 2000).

### Northern blot analysis

15 $\mu$g of total RNA isolated using Trizol reagent (Life Technologies, Inc), was size fractionated on a 1% agarose-formaldehyde gel in MOPS buffer and transferred to a MAGNA nylon membrane (MSI). The integrity of the RNAs was assessed by analysing the 18S and 28S ribosomal bands.

The human thymosin beta-10, 4F2 heavy chain, CD36, ATF-3 and c-kit cDNA probes of 500 bp were generated by a reverse transcription-polymerase chain reaction (RT-PCR). The primers were designed according to the published sequences. The probes were labelled with [α-32P] CTP using the multiprime DNA labelling system RPN.1601 (Amersham Pharmacia Biotech). Membranes were hybridised with the probes in ExpressHyb™ hybridisation solution (Clontech, CA, USA) according to manufacturer's directions. The membranes were exposed to Kodak X-ray film with an intensifying screen at −80˚C for autoradiography. The 18S and 28S bands were visualized on the blots by UV illumination and the 18S used for normalization of RNA loading. The autoradiograms were analysed by the Kodak Digital Science software and changes in expression calculated on the basis of relative band intensities.

### Western blot analysis

Cells were lysed in 20 nM Tris-acetate, pH 7.0, 0.27 M sucrose, 1 mM EDTA, 1 mM EGTA, 50 nM NaF, 1% Triton X-100, 5 mM Na-pyrophosphate, 10 mM Na-beta-glycerophosphate, 1 mM sodium orthovanadate, 10 $\mu$g ml$^{-1}$ aprotinin, and 10 $\mu$g ml$^{-1}$ PMSF. 5 $\mu$g of protein lysate was resolved by SDS-PAGE on 2–8% gradient gels (Invitrogen, the Nederlands). Proteins were transferred to PVDF membranes (Invitrogen, the Nederlands) and incubated in 5% non-fat dry milk in 10 mM Tris pH 7.5, 100 mM NaCl, 0.1% Tween for 1 h. Immunoblotting of primary antibody was performed in the same buffer overnight at 4˚C. Filters were extensively washed in 10 mM Tris pH 7.5, 100 mM NaCl, 0.1% Tween and bound antibodies were detected by incubation with alkaline-phosphatase conjugated secondary antibodies at 1:1000 for 1 h followed by washing and staining with a NBT/BCIP solution (Roche, Switzerland).

### RESULTS

### Gene expression profile of cell lines

RNA was isolated from the small-cell lung cancer cell line transfected with EGFRvIII (induced with doxycycline for 24 h or 48 h) or a vector control cell line (uninduced or induced with doxycycline for 48 h). The amplified, biotinylated RNA was hybridised to Affymetrix GeneChips, which were subsequently stained,

[1]Epidermal Growth Factor Receptor Mutation Type III Transfected Into a Small Cell Lung Cancer Cell Line Is Predominantly Localized at the Cell Surface and Enhance the Malignant Phenotype. Damstrup L, Pedersen MW, Bastholm L, Elling F, Poulsen HS (submitted).

 © 2001 Cancer Research Campaign scanned, and analysed using the GENECHIP 3.1 software. The intensities for gene transcripts (average differences) detected as present (P) represented a range spanning 3 orders of magnitude, from approximately 20 to the highest recorded value of 10 006 for ribosomal protein S2 (data not shown). However, the majority of the mRNAs detected had intensities of less than 1000. The genes associated with intensities greater than 1000 (≈ 400) include many of the ribosomal genes and commonly known high abundance genes such as tubulin, elongation factor 2 and ubiquitin. Interestingly, a number of cancer-related genes also had intensities above 1000 including: V-myc avian myelocytomatosis viral oncogene homologue, human pancreatic tumour-related protein, human tumour-associated 120 kDa nuclear protein (p120), and tumour protein, translationally controlled 1. Their high expression in the GLC3 cell lines suggests that they might play a role in the development of small-cell lung cancer.

To identify genes regulated by EGFRvIII a comparison was generated between the data sets from the GLC3-EGFRvIII induced with doxycycline for 24 h or 48 h and the vector control either uninduced or induced with doxycycline for 48 h. The level of gene expression of control cells after 48 h of doxycycline induction did not change.

Approximately 800 genes were identified whose mRNA expression levels were changed in the EGFRvIII expressing cells as compared to the control stimulated for 48 h with doxycycline. The majority of these (> 80%) represented genes for which the degree of change was less than 2-fold. 225 genes had a fold change (ratio of average differences) of 2 or more and had average difference changes (the difference between the EGFRvIII expressing cells and the control average differences) of more than 50 in either of the two time points as compared to the control. 188 genes had altered expression upon induction of EGFRvIII expression for 24 h and 175 after 48 h of induction as compared to the control. The majority of these 150 have similar expression at the two time points.

These genes were crudely divided into 13 categories on the basis of the cellular function of the protein they encode (transcriptional regulation, cytoskeletal and adhesion, metabolic, receptors and ligands, cell cycle, signal transduction, ribosomal, nuclear, translational regulation, apoptosis, immune-related, cancer-related, and miscellaneous). Due to space limitations only 9 of the categories are shown in Table 1.

It was not possible to detect EGFRvIII transcripts using the probe set for the EGFR precursor (X00588). The EGFR precursor mRNA extends 1701 bases longer in the 3′ end compared to the processed EGFRvIII mRNA. The 20 probes for detection of the EGFR precursor on the chip are designed to hybridise to sequences in this area and therefore cannot recognize EGFRvIII mRNA.

**Table 1** List of genes whose expression changed more than 2-fold between EGFRvIII- and control cells sorted on the basis of their cellular function: Transcriptional regulation (**A**), cytoskeletal or adhesion (**B**), receptors and ligands (**C**), cell cycle (**D**), apoptosis (**E**), signal transduction (**F**), and cancer related (**G**). The first 2 columns show the fold change in the gene expression between the vector controls and the doxycycline-induced (24 h or 48 h) EGFRvIII-expressing cells. At the bottom of each panel is an explanation of the colour codes used. The third and fourth columns describe the genes expressed and their Genbank accession. The absolute call states whether the gene transcripts are present (P), marginally present (M) or absent (A) in the experimental file. Difference call is a call of the change either decreased (D), increased (I), no change in (NC), marginally decreased (MD), or marginally increased expression (MI) of a particular gene. Average difference change (Avg. Diff. Change) is a measure of the change of intensities in expression of a particular gene

**A**

Transcriptional Regulation

| Fold Change 24th. | Fold Change 48th. | GenBank | Description | Absolute Call 24th. | Absolute Call 48th. | Diff.Call 24th. | Diff.Call 48th. | Avg.Diff.Change 24th. | Avg.Diff.Change 48th. |
|---|---|---|---|---|---|---|---|---|---|
| - 16.0 | - 16.0 | U22376 | c-myb gene extracted from Human (c-myb) “gene” complete primary “cds,” | P | P | D | D | -157 | -157 |
| - 12.7 | - 12.7 | L37868 | “Homo sapiens POU-domain transcription factor ““(N-Oct-3),”” complete cds” | M | M | D | D | -123 | -123 |
| - 6.4 | - 3.7 | M93119 | Human zinc-finger DNA-binding motifs (IA-1) “mRNA,”complete cds | A | P | D | D | -138 | -88 |
| - 4.1 | - 4.1 | X82125 | H. sapiens HOK-2 mRNA for zinc finger protein | A | A | D | D | -128 | -128 |
| - 3.8 | | L23959 | Homo sapiens E2F-related transcription factor (DP-1) “mRNA,” complete cds | A | P | D | NC | -79 | 25 |
| - 2.9 | - 2.5 | M88163 | Human global transcription activator homologous sequence “mRNA,” complete cds | A | P | D | D | -55 | -50 |
| - 2.7 | - 2.7 | X52611 | Human mRNA for transcription factor AP-2 | A | A | D | D | -127 | -127 |
| - 2.6 | | M16937 | Human homeo box c1 “protein,” “mRNA,” complete cds | A | P | D | NC | -55 | -15 |
| - 2.4 | - 2.4 | U80017 | Human basic transcription factor 2 p44 (btf2p44) gene, partial cds | A | A | D | D | -59 | -59 |
| - 2.3 | - 2.3 | Z11933 | “H.sapiens mRNA for N-Oct ““3,”” ““N-Oct5a,”” and N-Ocy 5b proteins” | A | A | D | D | -62 | -62 |
| - 2.2 | - 2.2 | D85425 | “Human mRNA for transactivator ““HSM-1,””complete cds” | P | P | D | D | 99 | -99 |
| - 2.2 | - 2.2 | X70683 | H.sapiens mRNA for SOX-4 protein | P | P | D | D | -722 | -722 |
| - 2.1 | | U07559 | Human ISL-1 (Islet-1) “mRNA, “complete cds | A | P | D | NC | -71 | -51 |
| | - 2.0 | HG3566 | Zinc Finger Protein (Gb:M88359) | A | M | NC | D | -9 | -153 |
| | - 2.4 | U32849 | Human Hou “mRNA,” compete cds | P | P | NC | I | 43 | 73 |
| | - 2.0 | JO5249 | Human replication protein A 32-kDa subunit “mRNA,: complete cds | P | P | I | I | 218 | 316 |
| - 2.0 | | AF005887 | Homo sapiens ATF family membe ATF6 (ATF6) “mRNA,” | A | P | NC | NC | 67 | 41 |
| - 2.0 | - 2.0 | X56681 | Human junD mRNA | P | P | I | I | 1098 | 1098 |
| - 2.0 | - 2.0 | M77698 | Homo sapiens GLI-Krupple related protein (YY1) “mRNA,” complete cds. | P | P | I | I | 234 | 229 |
| - 2.1 | - 2.1 | U96915 | Homo sapiens sin 3 associated polypeptide p18 (SAP18) “mRNA, “complete cds. | P | P | MI | MI | 184 | 184 |
| - 2.4 | | M31627 | Human X box binding protein-1 (XBP-1) “mRNA,’ comlpete cds | P | P | I | I | 331 | 207 |
| - 3.1 | - 2.6 | U49857 | Human transcriptional activator “mRNA,: complete cds | A | P | NC | MI | 74 | 55 |
| - 4.8 | - 4.6 | U35048 | Human TSC-22 protein “mRNA” complete cds | P | P | I | I | 491 | 468 |
| - 4.9 | - 3.1 | HG2724 | Oncogene “Tis/Chop, “Fusion Acticvated | P | P | I | I | 1360 | 756 |
| - 8.1 | - 5.6 | L198871 | Human activating transcription factor 3 (ATF3) “mRNA,” complete cds | P | P | NC | I | 202 | 220 |

| >5 | >3 | >2 | <2 | >-2 | <-2 | <-3 | -<5 |
|---|---|---|---|---|---|---|---|

Fold Induction — Fold Repression

B

| Cytoskeletal or Adhesion | | | | | | | | | |
|---|---|---|---|---|---|---|---|---|---|
| Fold Change 24th. | 48th. | GenBank | Description | Absolute Call 24th. | 48th. | Diff.Call 24th. | 48th. | Avg.Diff.Change 24th. | 48th. |
| - 16.3 | - 16.3 | M98399 | "Human antigen CD36 (clone 21) "mRNA," complete cds" | P | P | D | D | -161 | -161 |
| - 6.5 | - 12.9 | AB000114 | Human mRNA for "osteomodulin," complete cds | A | A | NC | D | -156 | -571 |
| - 5.8 | - 5.8 | L33930 | "Homo sapiens CD24 signal transducer "mRNA," | P | P | D | D | -250 | -250 |
| - 4.7 | - 4.7 | X698819 | H.sapiens ICAM-3 mRNA | A | A | D | D | -153 | -153 |
| - 4.0 | | D14678 | Human mRNA for kinesin-related "protein," partial cds | A | A | D | NC | -84 | -36 |
| - 3.0 | - 3.0 | Z24727 | H.sapiens tropomyosin isoform "mRNA," complete CDS | P | P | D | D | -615 | -615 |
| - 3.0 | - 2.6 | U03057 | Human actin bundling protein (HSN) "mRNA," complete cds | A | P | D | D | -348 | -320 |
| - 2.9 | - 2.0 | M82809 | Human annexin IV (ANX4) "mRNA," complete cds | P | P | D | D | -416 | -310 |
| - 2.8 | - 2.8 | X05608 | Human gene for neurofilament subunit NF-L | A | A | D | D | -73 | -73 |
| - 2.7 | - 2.7 | U46006 | "Human smooth muscle LIM protein (h-SmLIM) "mRNA," | P | P | D | D | -140 | -140 |
| - 2.5 | - 2.5 | M19267 | "Human tropomyosin ""mRNA," complete cds" | P | P | D | D | -568 | -568 |
| - 2.4 | | M61916 | Human laminin B1 chain "mRNA," complete cds | A | P | D | NC | -101 | -61 |
| - 2.4 | | L33477 | Human (clone 8B1) Br-cadherin "mRNA complete cds | P | P | D | NC | -196 | -121 |
| - 2.3 | - 2.3 | X54304 | Human mRNA for myosin regulatory light chain | A | A | D | D | -53 | -53 |
| - 2.3 | - 2.3 | X98801 | H.sapiens mRNA for dynactin | A | A | D | D | -146 | -146 |
| - 2.3 | - 2.3 | M55210 | Human laminin B2 chain (LAMB) gene | A | P | D | D | -94 | -81 |
| - 2.2 | - 2.2 | Z32765 | H.sapiens CD36 gene exon 15/gb=Z32765/ntype=DNA/annot=exon | P | P | D | D | -407 | -407 |
| - 2.1 | - 2.3 | HG2442 | Tropomyosin, "Alpha," "Muscle," Alt. Splice "2," Skeletal Muscle (Fibroblast) | A | P | D | NC | -60 | -64 |
| | - 2.5 | L19783 | Human GPI-H "mRNA," complete cds | P | P | NC | I | 57 | 120 |
| - 2.2 | - 2.4 | L04733 | Homo sapiens kinesin light chain "mRNA," complete cds | P | P | NC | I | 126 | 129 |
| - 2.3 | - 2.3 | X06956 | Homo HALPA44 gene for ""alpha-tubulin,""exons 3-Jan" | P | P | I | I | 135 | 135 |
| - 2.5 | - 2.5 | J04029 | "Homo sapiens keratin 10 type I intermediate filament (KRT10) ""mRNA | P | P | I | I | 640 | 640 |
| - 3.1 | - 3.5 | U20582 | "Human actin-like peptide "mRNA, "partial cds | P | P | NC | I | 230 | 283 |
| - 3.2 | - 4.7 | U21128 | Human lumican "mRNA," comlete cds | P | P | MI | I | 56 | 74 |
| - 3.8 | - 2.8 | D21255 | Human mRNA for "OB-cadherin-2,"complete cds | P | P | I | I | 79 | 85 |

| >5 | >3 | >2 | <2 | >-2 | <-2 | <-3 | <-5 |
|---|---|---|---|---|---|---|---|
| | Fold Induction | | | | | Fold Repression | |

C

| Receptors and Ligands | | | | | | | | | |
|---|---|---|---|---|---|---|---|---|---|
| Fold Change 24th. | 48th. | GenBank | Description | Absolute Call 24th. | 48th. | Diff.Call 24th. | 48th. | Avg.Diff.Change 24th. | 48th. |
| - 6.1 | - 6.1 | X06182 | Human c-kit proto-oncogne mRNA | P | P | D | D | -543 | -543 |
| - 3.4 | | M21551 | Human neuromedin B mRNA complete cds. | A | A | D | NC | -67 | -22 |
| - 2.8 | - 2.8 | U19247 | Human inferferon-gamma receptor alpha chain gene | P | P | D | D | -126 | -126 |
| - 2.2 | - 2.2 | S77410 | "type1 angiotensin II receptor""[human,""liver,""mRNA,""2268 nt]" | A | A | D | D | -57 | -57 |
| - 2.1 | - 2.1 | X98248 | H.sapiens mRNA for sortilin | P | P | D | D | -237 | -237 |
| | - 7.2 | M60278 | Human heparin-binding EGF-like growth factor "mRNA," complete cds | A | A | NC | D | -48 | -123 |
| | - 2.0 | D13641 | Human mRNA for KIAA0016 "gene," complete cds | P | P | D | D | -256 | -287 |
| | 2.2 | L38487 | Human estrogen receptor-related protein (hERRa1) "mRNA,"3' "end," | P | P | NC | I | 95 | 184 |
| | 2.1 | L10373 | Human (clone CCG-B7) mRNA sequence | A | P | NC | I | 18 | 55 |
| 3.1 | 2.8 | HG3415 | Poliovirus Receptor | P | P | MI | MI | 99 | 86 |
| 3.9 | 3.9 | J05200 | "Human ryanodine receptor ""mRNA," complete cds" | P | P | MI | MI | 66 | 66 |

D

| Cell Cycle | | | | | | | | | |
|---|---|---|---|---|---|---|---|---|---|
| Fold Change 24th. | 48th. | GenBank | Description | Absolute Call 24th. | 48th. | Diff.Call 24th. | 48th. | Avg.Diff.Change 24th. | 48th. |
| - 3.6 | - 3.6 | | "Human butyrophilin (BTF4)" "mRNA," "complete cds" | A | A | D | D | -115 | -115 |
| - 2.3 | - 2.3 | | "Human butyrophilin (BTF4)" "mRNA," "complete cds" | P | P | D | D | -276 | -276 |
| - 2.1 | - 2.1 | | Human mRNA for cyclin A | A | A | D | D | -125 | -125 |
| - 2.1 | | | Human Hs-cul-1 "mRNA," comlete cds | P | P | D | D | -387 | -273 |
| 2.1 | 3.3 | | Human thymidylate kinase (CDC8) "mRNA," complete cds | P | P | NC | MI | 100 | 110 |
| 10.6 | 10.6 | | G0S2 gene extracted from Human GOS2 "gene," 5' flank and cds | P | P | MI | MI | 101 | 101 |

*© 2001 Cancer Research Campaign*

E

| Apoptosis | | | | | | | | | |
|---|---|---|---|---|---|---|---|---|---|
| Fold Change 24th. | Fold Change 48th. | GenBank | Description | Absolute Call 24th. | Absolute Call 48th. | Diff.Call 24th. | Diff.Call 48th. | Avg.Diff.Change 24th. | Avg.Diff.Change 48th. |
| - 4.2 | - 3.8 | D82345 | Human mRNA for NB thymosin “beta,” complete cds | P | P | D | D | -394 | -359 |
| - 2.6 | - 2.6 | S54005 | “thymosin beta,-10” “[human,” “metastic melanoma cell ““line, “mRNA | P | P | D | D | -1411 | -1411 |
| | - 2.3 | L08246 | Human myeloid cell differentiation protein (MCL1) mRNA | A | P | NC | I | 50 | 100 |

| >5 | >3 | >2 | <2 | >-2 | <-2 | <-3 | <-5 |
|---|---|---|---|---|---|---|---|

Fold Induction Fold Repression

F

| Signal Transduction | | | | | | | | | |
|---|---|---|---|---|---|---|---|---|---|
| Fold Change 24th. | Fold Change 48th. | GenBank | Description | Absolute Call 24th. | Absolute Call 48th. | Diff.Call 24th. | Diff.Call 48th. | Avg.Diff.Change 24th. | Avg.Diff.Change 48th. |
| - 3.5 | -2.8 | M31158 | Human cAMP-dependant protein kinase subunit RII-beta “mRNA,” | A | P | D | D | -80 | -72 |
| - 2.3 | - 2.3 | X60673 | Human AK3 mRNA for adenylate kinase 3. | A | A | D | D | -174 | -174 |
| - 2.2 | | M15205 | Human thymidane kinase “mRNA,” complete cds | A | P | D | NC | -145 | -36 |
| - 2.1 | | U09564 | Human serine kinase “mRNA,” comlpete cds | P | P | D | MD | -351 | -314 |
| - 2.0 | - 2.0 | X89416 | H.sapiens mRNA for protein phosphatase 5 | A | A | D | D | -216 | -216 |
| | - 2.0 | M86699 | Human kinase (TTK) “mRNA,” complete cds | P | P | NC | MD | -113 | -126 |
| 2.1 | 2.4 | S74221 | IK=IK factor “[human,” leukemic cells “K562,” chronic myeloid leukemia | P | P | NC | I | 80 | 99 |
| 2.3 | | U07563 | Human proto-oncogene tyrosine-protein kinase (ABL) gene, | P | P | NC | I | 144 | 122 |
| 2.3 | 2.3 | X68277 | H.sapiens CL 100 mRNA for protein tyrosine phosphatase | P | P | I | NC | 55 | 55 |
| 6.4 | 5.1 | U27655 | Human RGP3 “mRNA,” complete cds | A | P | NC | I | 142 | 108 |

G

| Cancer Related | | | | | | | | | |
|---|---|---|---|---|---|---|---|---|---|
| Fold Change 24th. | Fold Change 48th. | GenBank | Description | Absolute Call 24th. | Absolute Call 48th. | Diff.Call 24th. | Diff.Call 48th. | Avg.Diff.Change 24th. | Avg.Diff.Change 48th. |
| - 4.5 | - 2.3 | L13210 | Human Mac-2 binding protein mRNA,” complete cds | A | P | D | NC | -356 | -268 |
| - 3.7 | - 2.2 | M11717 | Human heat shock protein (hsp 70) gene, complete cds | A | P | D | NC | -82 | -60 |
| - 3.2 | - 2.4 | U17714 | Human putative tumor suppressor (SNC6) “mRNA,” comlete cds | A | A | D | D | -56 | -46 |
| - 2.0 | - 2.5 | U10686 | Human MAGE-11 antigen (MAGE11) “gene.” complete cds | A | P | D | NC | -99 | -117 |
| | - 2.3 | L02785 | Homo sapiens colon mucosa-associated (DRA) “mRNA, complete cds | A | A | NC | D | -11 | -64 |
| 2.5 | 2.1 | M80244 | Human E16 “mRNA, “complete cds | P | P | I | I | 802 | 570 |
| 3.5 | 3.0 | M21904 | Human 4F2 glycosylated heavy chain (4F2HC) antigen gene | P | P | MI | I | 1009 | 809 |
| 4.2 | 4.2 | X75308 | H.sapiens mRNA for collagenase 3 | P | P | I | I | 130 | 130 |

| >5 | >3 | >2 | <2 | >-2 | <-2 | <-3 | <-5 |
|---|---|---|---|---|---|---|---|

Fold Induction Fold Repression

As can be seen on Table 1 (A) the expression of many genes encoding transcription factors were altered in the EGFRvIII transfected cell line induced for both 24 and 48 h as compared to the vector control induced for 48 h. These included the genes for c-Myb, transcription factor AP-2, JunD, N-Oct 3, SOX-4, TSC-22 and activating transcription factor 3 (ATF-3).

Changes in expression of genes encoding cell adhesion molecules and structural proteins were also found (Table 1, B). For example, the cell adhesion molecules CD36, CD24 and ICAM-3 were decreased at both time points, while increased expression was observed for the gene encoding OB-cadherin-2. The expression of different growth factors and receptors were also altered. A decrease in the level of mRNAs encoding the Kit receptor, heparin-binding EGF-like growth factor, interferon-gamma receptor alpha chain, were observed (Table 1, C).

In the GLC3-EGFRvIII the level of only a few mRNAs encoding signal transduction molecules was altered. Most importantly an increase in mRNA levels was found for MAP Kinase Phosphatase 1 (MKP-1, CL100), (Table 1, F).

Amongst the category of genes related to cancer the mRNA levels of Mac-2 binding protein, Hsp70, MAGE-11 decreased at both time points, while the level of mRNAs encoding 4F2 light chain (E16), 4F2 heavy chain, and collagenase 3 were increased (Table 1, G).

### Verification of selected results by Northern blot analysis

In order to confirm changes in mRNA levels detected by the GeneChip analysis, Northern blot analyses were performed. 5 gene products were selected: c-kit, CD36, and thymosin beta 10 whose expression were decreased and 4F2 glycosylated heavy chain and ATF-3 whose expression were increased.

As can be seen in Figure 1A the c-kit mRNA level decreased in EGFRvIII transfected cell line by a factor of 6.8 (24 h) and 7.4 (48 h) as calculated by the band intensities on the Northern blot (6.1 and 6.1 by GeneChip analysis) respectively in the GLC3-EGFRvIII doxycycline-induced cells as compared to the control cells doxycycline induced for 48 hours.

The fold change in CD36 expression in Northern blot could not be calculated as no CD36 mRNA could be detected in the EGFRvIII-transfected cell line doxycycline induced for 24 or 48 h. The level of thymosin beta 10 was decreased by a factor of 6.3 and 10 (2.6 and

*© 2001 Cancer Research Campaign*

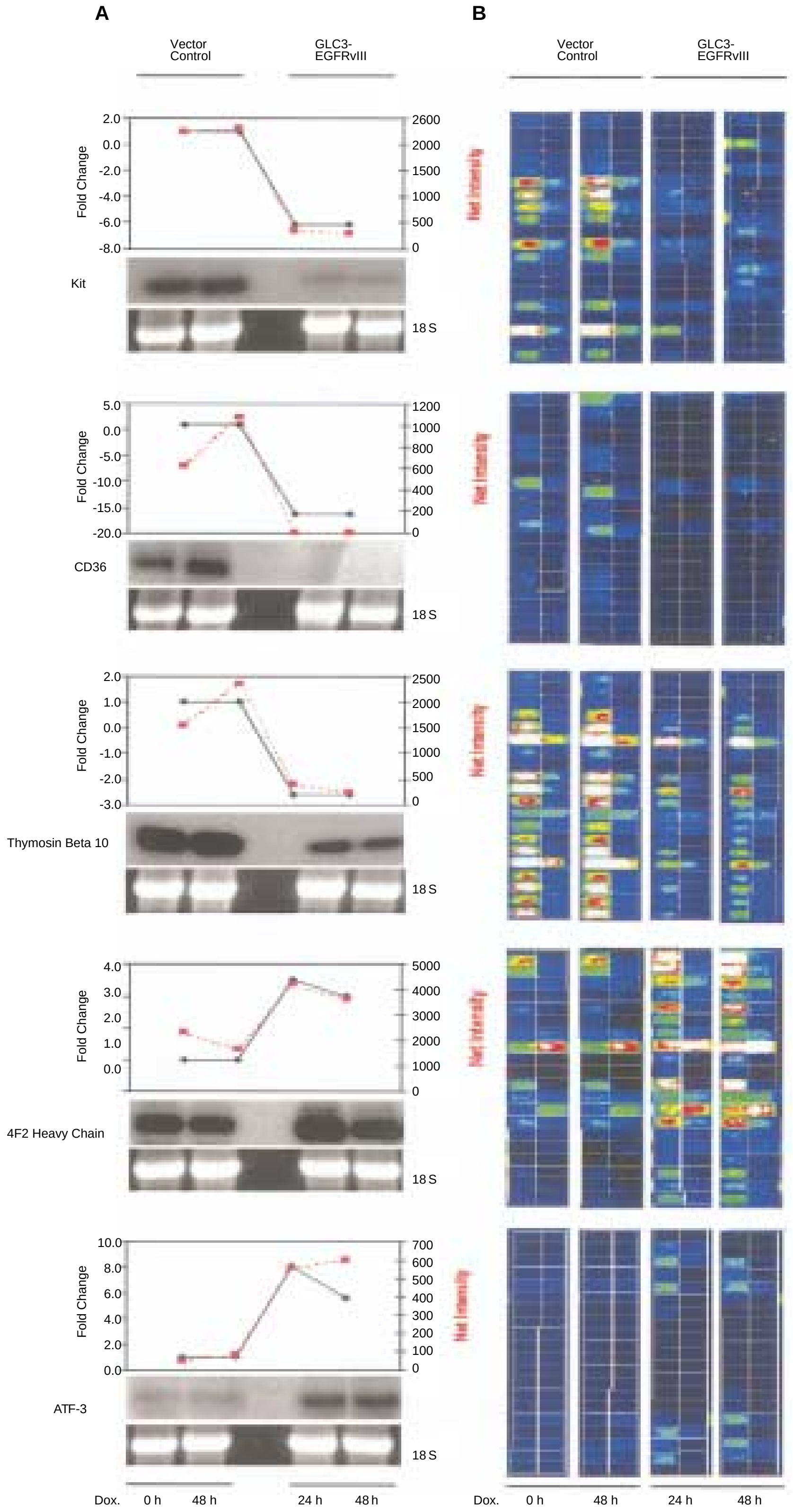

**Figure 1** Northern blot analyses (**A**) and corresponding hybridisation GeneChip results (**B**) of 5 selected genes that were differentially expressed in the GLC3-EGFRvIII cell line as compared to the vector control. The autoradiograms (middle) show the mRNA levels of c-kit, Thymosin Beta-10, CD36, 4F2 heavy chain and ATF-3 in the 4 cell samples. The first panel for each molecule shows a comparison between fold change measured by GeneChip (black) and net intensity of the bands generated by Northern blot analysis (red). The lower panels show the 18 S ribosomal bands visualised on the blots by UV light and were used as mRNA loading controls. Shown in panel B are the corresponding GeneChip hybridisation results, where white/red designates a high level of hybridisation while blue/black indicates a low level of hybridisation. The first row is the perfect match hybridisation and the second row the hybridisation to the corresponding mismatch oligonucleotide probes, used as subtraction for unspecific hybridisation

*© 2001 Cancer Research Campaign* 

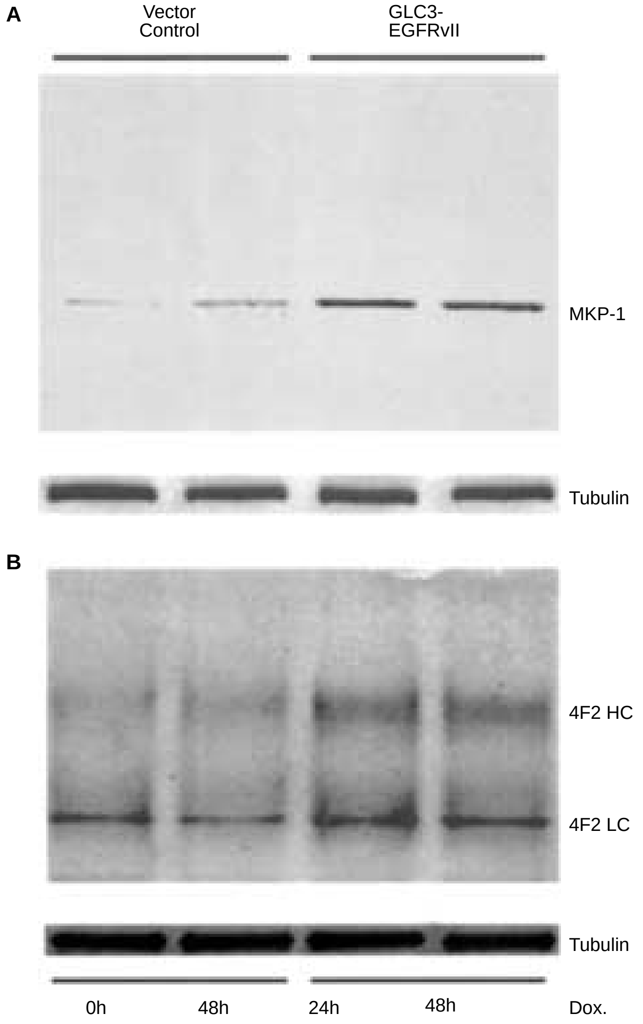

**Figure 2** 5 $\mu g$ aliquots of whole-cell lysates were processed for Western blot analysis. MKP-1 levels (**A**) were detected using rabbit polyclonal antibodies directed against MKP-1. The level of MKP-1 was only detectable in the GLC3-EGFRvIII cell line. CD98 (4F2 heavy and light chain) levels (**B**) were detected using rabbit polyclonal antibodies directed against CD98. The level of CD98 was higher in the EGFRvIII transfected cell line as compared to the vector controls. The amount of protein loaded being similar was in all cases verified using mouse monoclonal antibodies directed against human Tubulin

2.6 by GeneChip analysis) in the GLC3-EGFRvIII cell line as compared to the control cell line. The 4F2 heavy chain mRNA was increased by a factor of 3.1 and 2.7 (3.5 and 3.0 by GeneChip analysis) respectively in the GLC3-EGFRvIII doxycycline-induced cell as compared to the control cell doxycycline induced for 48 h. Finally the level of ATF-3 increased by factors of 7.5 and 7.6 (8.1 and 5.6 by GeneChip analysis).

### Verification of results by Western blot analysis

We performed Western blot analysis on 3 selected proteins in order to further validate our GeneChip data. CD98 (4F2 heavy chain and 4F2 light chain) and MKP-1 were chosen, as specific antibodies were commercially available.

A significant increase in the protein level of MKP-1 was observed, as seen in Figure 2A, in the EGFRvIII transfected cells when compared to the control cells. MKP-1 was barely detectable in the control cell line both uninduced and doxycycline-induced. Under the conditions of the Western blot analysis the CD98 dimer separates as a heavy chain and a light chain, 4F2HC and 4F2LC respectively which both are detected by the CD98 antibody. Shown in Figure 2B are the results from the Western blot analysis of CD98. The level of both 4F2LC and 4F2HC increased, although more pronounced for the latter, when comparing the EGFRvIII-expressing cells with the control cells.

### DISCUSSION

The role of the constitutively active EGFRvIII in the development and progression of cancers has been studied extensively over the past several years, and the ability of EGFRvIII to transform fibroblasts in vitro and confer enhanced tumorigenicity to cancer cells both in vitro and in vivo are supported by numerous studies (Garcia et al, 1993). However, little is known about the molecular mechanisms behind this ability of EGFRvIII, although EGFRvIII clearly induces a signal transduction network distinct from that of the ligand-activated EGFR (Antonyak et al, 1998; Moscatello et al, 1998; Lorimer and Lavictoire, 2001). Virtually nothing is known about genes whose expression is regulated by EGFRvIII. Thus to identify novel EGFRvIII-regulated genes, oligonucleotide arrays were used to study the changes in mRNA levels induced by expression of EGFRvIII in the GLC3-EGFRvIII cell line compared to a vector control. The use of oligonucleotide arrays allows simultaneous analysis of expression profiles of thousands of genes. To our knowledge this is the first comprehensive study of transcriptional changes related to EGFRvIII.

We identified 225 genes whose expression was up-or down-regulated by at least a factor of 2 in either of the two time points of induction used and the majority of these had similar expression at the two time points. The similarity of the expression results at the two time points correlate well with the constitutive nature of the EGFRvIII. The addition of doxycycline did not influence the level of gene expression in the control cells. A few of the results from the gene chip analysis were verified by Northern blot analysis and we found a strong qualitative correlation between oligonucleotide array hybridisation data and Northern blot analysis (Figure 1). Furthermore, to test the strength of the gene chip analysis, genes were selected that had increased expression (4F2 heavy chain and ATF-3) or decreased expression (c-kit, CD36 and thymosin beta 10) and either high level of expression (4F2 heavy chain, thymosin beta 10) or low level of expression (CD36 and ATF-3). For completeness a correlation between array expression results and changes in protein levels were confirmed for 4F2 heavy chain, 4F2 light chain and MKP-1.

Many of the differences in expression level between the EGFRvIII-expressing cell line and the control cell line makes sense. For example MKP-1 (CL100) has been proposed to mediate the cross talk between the constitutively active JNK signal transduction pathway and the Ras/Raf/ERK signal transduction pathway at the level of ERK, resulting in inactivation of the latter in EGFRvIII-transfected cells (Antonyak et al, 1998). We found an increased level of MKP-1 mRNA and protein in our EGFRvIII-expressing cells after induction of EGFRvIII expression thus supporting this hypothesis. In addition we found an increased expression of a number of other genes that are known to be

© 2001 Cancer Research Campaign 

regulated by active JNK which include ATF-3, collagenase 3 (MMP-13), and Jun D. Again this supports the finding from other EGFRvIII transfected cell lines that EGFRvIII utilises a constitutively active PI3K/JNK pathway (Antonyak et al, 1998; Moscatello et al, 1998).

We have previously found that the EGFRvIII-transfected GLC3 cell line had an increased invasive capacity in vitro as compared to the vector control. Interestingly the mRNA level of collagenase 3 was increased in these cells as compared to the vector control. Collagenase 3 expression has been documented in various cancers which are aggressive and invasive. In particular collagenase 3 expression was seen at the invading front (Brinckerhoff et al, 2000). Collagenase 3 therefore seems to play an important role in tumour invasion and one could speculate that there might be a correlation between the collagenase expression in the EGFRvIII-transfected cell line and their invasive potential. We are currently testing this hypothesis.

Our data demonstrate that amongst the 6800 human genes examined, the expression of 225 were altered more than 2-fold in either of two time points after induction of EGFRvIII expression. All of these represent potential EGFRvIII-regulated genes not previously described and include a number of genes encoding transcription factors, cell adhesion molecules, and other genes related to cancer. However, a significant portion of the genes have at present no known function. Our comprehensive data may prove useful in the future as many, or all, of these genes will be characterized. This will give further insight into the role of EGFRvIII in the malignant phenotype. We did not compare the changes in the EGFRvIII-transfected cell line with a EGFR-transfected SCLC cell line. This was not performed as EGFR-transfected cells were not available but most importantly, it was not clear which time point after EGF addition would be relevant to use for comparison. Furthermore, such a comparison would most likely have limited value as differences in the pathways used between EGFR and EGFRvIII are clearly described (Antonyak et al, 1998; Moscatello et al, 1998; Lorimer and Lavictoire, 2001). We are currently evaluating results in other EGFRvIII-expressing cell lines and in xenotransplanted tumours to clarify whether the results described herein are receptor or cell-type specific.

### REFERENCES

Antonyak MA, Moscatello DK and Wong AJ (1998) Constitutive activation of c-Jun N-terminal kinase by a mutant epidermal growth factor receptor. *J Biol Chem* **273**: 2817–2822

Batra SK, Castelino-Prabhu S, Wikstrand CJ, Zhu X, Humphrey PA, Friedman HS and Bigner DD (1995) Epidermal growth factor ligand-independent, unregulated, cell-transforming potential of a naturally occurring human mutant EGFRvIII gene. *Cell Growth Differ* **6**: 1251–1259

Brinckerhoff CE, Rutter JL and Benbow U (2000) Interstitial collagenases as markers of tumor progression. *Clin Cancer Res* **6**: 4823–4830

Der SD, Zhou A, Williams BR and Silverman RH (1998) Identification of genes differentially regulated by interferon alpha, beta, or gamma using oligonucleotide arrays. *Proc Natl Acad Sci USA* **95**: 15623–15628

Garcia dP, I, Adams GP, Sundareshan P, Wong AJ, Testa JR, Bigner DD and Weiner LM (1993) Expression of mutated epidermal growth factor receptor by non-small cell lung carcinomas. *Cancer Res* **53**: 3217–3220

Kaminski N, Allard JD, Pittet JF, Zuo F, Griffiths MJ, Morris D, Huang X, Sheppard D and Heller RA (2000) Global analysis of gene expression in pulmonary fibrosis reveals distinct programs regulating lung inflammation and fibrosis. *Proc Natl Acad Sci USA* **97**: 1778–1783

Lorimer IA and Lavictoire SJ (2001) Activation of extracellular-regulated kinases by normal and mutant EGF receptors. *Biochim Biophys Acta* **1538**: 1–9

Moscatello DK, Holgado-Madruga M, Godwin AK, Ramirez G, Gunn G, Zoltick PW, Biegel JA, Hayes RL and Wong AJ (1995) Frequent expression of a mutant epidermal growth factor receptor in multiple human tumors. *Cancer Res* **55**: 5536–5539

Moscatello DK, Holgado-Madruga M, Emlet DR, Montgomery RB and Wong AJ (1998) Constitutive activation of phosphatidylinositol 3-kinase by a naturally occurring mutant epidermal growth factor receptor. *J Biol Chem* **273**: 200–206

Nagane M, Coufal F, Lin H, Bogler O, Cavenee WK and Huang HJ (1996) A common mutant epidermal growth factor receptor confers enhanced tumorigenicity on human glioblastoma cells by increasing proliferation and reducing apoptosis. *Cancer Res* **56**: 5079–5086

Nagane M, Levitzki A, Gazit A, Cavenee WK and Huang HJ (1998) Drug resistance of human glioblastoma cells conferred by a tumor-specific mutant epidermal growth factor receptor through modulation of Bcl-XL and caspase-3-like proteases. *Proc Natl Acad Sci USA* **95**: 5724–5729

Nishikawa R, Ji XD, Harmon RC, Lazar CS, Gill GN, Cavenee WK and Huang HJ (1994) A mutant epidermal growth factor receptor common in human glioma confers enhanced tumorigenicity. *Proc Natl Acad Sci USA* **91**: 7727–7731

Olapade-Olaopa EO, Moscatello DK, MacKay EH, Horsburgh T, Sandhu DP, Terry TR, Wong AJ and Habib FK (2000) Evidence for the differential expression of a variant EGF receptor protein in human prostate cancer. *Br J Cancer* **82**: 186–194

Prigent SA and Lemoine NR (1992) The type 1 (EGFR-related) family of growth factor receptors and their ligands. *Prog Growth Factor Res* **4**: 1–24

Sugawa N, Yamamoto K, Ueda S, Morita N, Kita M, Nishino H, Fushiki S and Okabe T (1998) Function of aberrant EGFR in malignant gliomas. *Brain Tumor Pathol* **15**: 53–57

*© 2001 Cancer Research Campaign*
