# Profile of a differentially expressed genes mediated by the type III epidermal growth factor receptor mutation expressed in a small-cell lung cancer cell line

**DOI:** 10.1054/bjoc.2001.2199

**Published:** 2001-12


					2023
British Journal of Cancer (2001) 85(12), 2023-2025
(c) 2001 Cancer Research Campaign
doi: 10.1054/ bjoc.2001.2199, available online at http://www.idealibrary.com on http://www.bjcancer.com
Profile of differentially expressed genes mediated by the type III epidermal growth factor receptor mutation
expressed in a small-cell lung cancer cell line
MW Pedersen, T Thykjaer, TF Orntoft, L Damstrup and HS Poulsen
Br J Cancer 85(8): 1211-1218
Table 1 was published incorrectly, and is reproduced correctly below. The publishers wish to apologise for this error.
Erratum
Table 1 List of genes whose expression changed more than 2-fold between EGFRvIII- and control cells sorted on the basis of their cellular function:
Transcriptional regulation (A), cytoskeletal or adhesion (B), receptors and ligands (C), cell cycle (D), apoptosis (E), signal transduction (F), and cancer related
(G). The first 2 columns show the fold change in the gene expression between the vector controls and the doxycycline-induced (24 h or 48 h) EGFRvIII-
expressing cells. At the bottom of each panel is an explanation of the colour codes used. The third and fourth columns describe the genes expressed and their
Genbank accession. The absolute call states whether the gene transcripts are present (P), marginally present (M) or absent (A) in the experimental file.
Difference call is a call of the change either decreased (D), increased (I), no change in (NC), marginally decreased (MD), or marginally increased expression
(MI) of a particular gene. Average difference change (Avg. Diff. Change) is a measure of the change of intensities in expression of a particular gene
2024 MW Pedersen et al
British Journal of Cancer (2001) 85(12), 2023-2025 (c) 2001 Cancer Research Campaign
Erratum 2025
British Journal of Cancer (2001) 85(12), 2023-2025
(c) 2001 Cancer Research Campaign

				

## 

**Correction to:** *British Journal of Cancer* (2001) **85**, 1211–1218. doi:10.1054/bjoc.2001.2053

